# The role of ticagrelor in acute ischaemic stroke and high-risk TIA management

**DOI:** 10.1007/s00415-020-10336-1

**Published:** 2020-12-23

**Authors:** Claire MacIver, Tom Hughes

**Affiliations:** Department of Neurology, University Hospital of Wales, Heath Park, Cardiff, CF14 4XN UK

Following a transient ischaemic attack (TIA) or cerebral infarction associated with mild disability (NIHSS ≤ 5), the risk of recurrence is high in the subsequent 30 days, highlighting the importance of early interventions. Current regimens support the use of one antiplatelet agent in infarction with severe disability, typically Aspirin (a prostaglandin H synthase in inhibitor) 300 mg daily for 2 weeks, followed by Clopidogrel (which inhibits the binding of adenosine diphosphate to its platelet P2Y12 receptor) 75 mg daily. For patients with high-risk TIAs or cerebral infarction with mild disability (NIHSS score of five or less), dual-antiplatelet therapy for 10–21 days (depending on bleeding risk) with aspirin 75 mg and clopidogrel 75 mg is recommended, followed by clopidogrel 75 mg alone.

A disadvantage of clopidogrel is that in a significant minority of patients, activation via hepatic pathways is ineffective, with a corresponding reduction in antiplatelet activity. Ticagrelor (which prevents ADP-mediated P2Y12-dependent platelet activation) is not subject to such variation in metabolism, and has been proposed as an alternative.

We have reviewed three papers describing three different comparisons in symptomatic patients with cerebrovascular disease: ticagrelor in patients with and without prior use of aspirin; ticagrelor and aspirin with aspirin; ticagrelor and aspirin with aspirin and clopidogrel. The difference ticagrelor makes does not appear to be dramatic. Overall, the evidence does suggest that we should not yet remove aspirin (bought for pennies from the local pharmacist)—from our rucksacks, glove compartments and kitchen pharmacies.

## Efficacy and safety of ticagrelor in relation to aspirin use within the week before randomisation in the SOCRATES trial

This paper was based on a pre-specified subgroup analysis of SOCRATES (aspirin and ticagrelor in acute ischaemic stroke and TIA), which examined patients in whom aspirin had been used in 7 days before randomisation. Patients with ischaemic infarction with mild disability (NIHSS ≤ 5) or high-risk TIA (ABCD^2^ score ≥ 4 or symptomatic ipsilateral intra- or extra-cranial stenosis) were randomised (1:1) within 24 h to either high-dose aspirin or ticagrelor. ABCD^2^ score is a clinical risk score following TIA, based on age, blood pressure, clinical features, duration of symptoms, and diabetes. The main outcome measure was stroke, myocardial infarction (MI) or death within 90 days of randomisation. Major bleeding was a secondary outcome measure.

4232 of the 13,199 patients met the criteria for prior-aspirin use; 2130 were randomised to ticagrelor treatment, 2102 to more aspirin. 8967 patients did not have prior-aspirin use; 4459 were randomised to ticagrelor, 4508 to aspirin.

In the prior-aspirin group, stroke, MI or death at 7 days occurred in 3.8% in those given ticagrelor versus 5.1% in those given more aspirin (*p* = 0.04). After 90 days, this was 6.5% of those given ticagrelor versus 8.3% in those given more aspirin (*p* = 0.02). In those who did not take aspirin during the week before randomisation, stroke, MI or death at 7 occurred in 3.9% given ticagrelor, and 4.6% for those given aspirin (*p* = 0.1); at 90 days, this was 6.9% for ticagrelor and 7.1% for aspirin (*p* = 0.59). At 90 days, there was no significant difference in prior-aspirin treatment effect (*p* = 0.1). There was a non-significant increase in major bleeding in the ticagrelor arm among the prior-aspirin treated cohort compared to those who continued on aspirin (0.7% compared to 0.4%, *p* = 0.28).

*Comments*: This study did not show any significant benefit in those who had used aspirin during the week before randomisation, with a non-significant trend towards an increase in adverse events. Note should be made that this was an exploratory secondary analysis rather than the primary analysis. The short-lived use of aspirin in this study, without undertaking prolonged dual-antiplatelet therapy, limits the conclusions that can be drawn about the possible effects of a longer period of dual therapy. In addition, the study cohorts are not likely to be representative as the prior-aspirin cohort had a different risk factor profile, with a higher atherosclerotic burden and higher rates of ipsilateral carotid atherosclerosis. Participants already on aspirin may have made lifestyle modifications before randomisation and/or be less likely to make further lifestyle alterations following the event that involved them in the trial.

In the context of these limitations, this study offers weak evidence to give ticagrelor rather than aspirin to patients who have used aspirin in the 7 days before they present with a TIA or cerebral infarction with mild disability.

*Wong et al. (2018). Stroke. 49(7):1678–1685*.

## Ticagrelor and aspirin or aspirin alone in acute ischaemic stroke or TIA

The THALES study is a randomised, placebo-controlled trial involving 414 centres in 28 countries across Europe, Asia and America, that compared aspirin alone to aspirin and ticagrelor. 11,016 participants with non-cardioembolic cerebral infarction and mild disability (NIHSS score ≤ 5), or high-risk TIA (ABCD^2^ score ≥ 6) were randomised. Primary outcome was stroke or death within 30 days, and secondary outcomes were first subsequent ischaemic stroke and disability at 30 days.

There was a significant difference in stroke or death within 30 days; 6.6% in the aspirin-only group and 5.5% in the aspirin and ticagrelor group (*p* = 0.02). There was no significant difference in disability at 30 days between these groups. There was also a significant increase in severe bleeding in the combination therapy group (0.5% compared to 0.1% with aspirin alone, *p* = 0.001), which remains significant following Bonferroni correction for multiple comparisons.

*Comment*: This study suggests a benefit of combination therapy with aspirin and ticagrelor in patients with mild-to-moderate non-cardioembolic cerebral infarction compared to aspirin alone, but with an increased bleeding risk. The number needed to treat was 92 in this study, with number needed to harm for severe bleeding of 263. This study, therefore, offers evidence to support combination therapy, although it is unclear whether the risk of bleeding is acceptable. It is important to note that patients who underwent thrombolysis and thrombectomy were excluded, which may restrict the generalisability of study results. In addition, only 0.5% of participants were from Black ethnic groups, which may be a significant limitation as Clopidogrel is ineffective in a high proportion of Black, Asian and minority ethnic group individuals.

*Johnson *et al. *(2020). The New England Journal of Medicine 383(3):207–217.*

## Ticagrelor plus aspirin verses clopidogrel plus aspirin for platelet reactivity in patients with minor stroke or transient ischaemic attack: open label, blind endpoint, randomised controlled phase II trial

This was a randomised, blind-endpoint phase II trial across 26 centres in China. It compared ticagrelor with aspirin to clopidogrel with aspirin in cerebral infarction associated with mild disability (NIHSS ≤ 3) and moderate to high-risk TIA (ABCD^2^ score ≥ 4 or ≥ 50% stenosis of cervical or intracranial vessels) within 24 h of presentation. The proportion of participants with high platelet reactivity at 90 days was the primary outcome. Secondary outcomes included the proportion of participants with high platelet reactivity in genetic subtypes affecting clopidogrel metabolism, recurrent stroke, and composite vascular events at 90 days, 6 months and 1 year. The primary safety outcome was major bleeding events. The study was terminated after a pre-specified interim analysis showed a pre-specified *p* value of ≤ 0.005 in the primary outcome.

Of 675 patients, 336 were randomised to ticagrelor with aspirin treatment (280 were included in the primary outcome analysis), and 339 to clopidogrel with aspirin (290 included in the primary outcome analysis). Those randomised but not treated within the specified 24-h window were removed from the analysis.

The ticagrelor with aspirin group had significantly lower rates of high platelet reactivity than the clopidogrel with aspirin group (12.5% vs 29.7%, *p* < 0.001), finding a non-significant difference in recurrent cerebral infarction (6.3% vs 8.8%, *p* = 0.2), and in composite vascular events. There was also a significant reduction in subsequent cerebral infarction in the ticagrelor group (from 13.1% to 6%, *p* = 0.04) in those individuals with large artery atherosclerosis.

Following subgroup analysis, CYP2C19 carriers (associated with a reduced effect of clopidogrel), had a significant reduction in 90-day high platelet reactivity from 35.4% in the clopidogrel group to 10.8% in the ticagrelor group (*p* < 0.001). There was no significant difference in major bleeding between the two groups. Of note, more patients discontinued treatment in the ticagrelor arm, because of side effects including epistaxis and dyspnoea.

*Comments*: This study demonstrated a non-significant trend towards a reduction in risk of further stroke at 90 days in patients taking ticagrelor with aspirin rather than ticagrelor with clopidogrel. The lower rate of high platelet reactivity is of interest, but in the absence of a clinically meaningful difference in a relevant outcome measure it remains unclear whether ticagrelor should be used as an alternative to clopidogrel in clinical practice.

Some caution is also required in the interpretation of results regarding the 24 pre-specified secondary outcome measures since there is a lack of effective correction for multiple comparisons. In addition, the differences in clopidogrel metabolism in different ethnic groups means that results may not be applicable to cohorts of non-Chinese ethnicities.

*Wang *et al. *(2019). BMJ. 365:l2211(1–11).*

